# Effects of Periprocedural Tirofiban vs. Oral Antiplatelet Drug Therapy on Posterior Circulation Infarction in Patients With Acute Intracranial Atherosclerosis-Related Vertebrobasilar Artery Occlusion

**DOI:** 10.3389/fneur.2020.00254

**Published:** 2020-04-15

**Authors:** Xuan Sun, Huijun Zhang, Xu Tong, Feng Gao, Gaoting Ma, Zhongrong Miao

**Affiliations:** ^1^Department of Interventional Neuroadiology, Beijing Tiantan Hospital, Capital Medical University, Beijing, China; ^2^Department of Neurology, Tong Ren Hospital Shanghai Jiaotong University School of Medicine, Shanghai, China

**Keywords:** endovascular treatment, tirofiban, antiplatelet, intracranial atherosclerosis-related vertebrobasilar artery occlusion, vertebrobasilar artery occlusion

## Abstract

**Background and purpose:** Tirofiban and oral antiplatelet drugs can be used to inhibit reocclusion and restore microvascular reperfusion during endovascular treatment (EVT). This study compared recanalization rates, symptomatic intracranial hemorrhage (SICH), 90 day mortality, and functional outcomes between periprocedural tirofiban and antiplatelet therapy in patients with acute intracranial atherosclerosis-related vertebrobasilar artery occlusion.

**Methods:** A total of 105 consecutive patients with acute intracranial atherosclerosis-related vertebrobasilar artery occlusion who underwent EVT + tirofiban + oral antiplatelet or EVT + oral antiplatelet therapy at the Beijing Tiantan Hospital between January 2012 and July 2018 were included. Baseline characteristics, procedural parameters, and functional outcomes were assessed.

**Results:** Among the 105 patients, 74 underwent EVT + tirofiban + oral antiplatelet therapy, while 31 underwent EVT + oral antiplatelet drug therapy. EVT + tirofiban + oral antiplatelet therapy resulted in higher recanalization rates compared to EVT + oral antiplatelet drug therapy (93.24% vs. 77.42%; *p* = 0.038), whereas the risk for SICH, 90 day mortality, and functional independence outcomes did not differ between the groups. Logistic regression analysis revealed that EVT + tirofiban + oral antiplatelet therapy had an increased probability of higher recanalization rates (OR 0.18 [95% confidence interval (CI) 1.24–24.39]; *p* = 0.025). There were no differences in SICH (OR 0.00 [95% CI 0.00–Inf]; *p* = 0.998), 90 day mortality (OR 1.19 [95% CI 0.17–4.05]; *p* = 0.826), or functional independence (modified Rankin score 0 to ≤ 2) (OR 1.43 [95% CI 0.23–2.17]; *p* = 0.538) between the groups.

**Conclusions:** Ninety day functional outcomes of EVT + tirofiban + oral antiplatelet therapy were not superior to those of EVT + oral antiplatelet drug therapy; however, the recanalization rate was higher and the risks for SICH and 90 day mortality were lower.

## Introduction

Many randomized trials have demonstrated the improved efficacy of endovascular treatment (EVT) compared with standard medical care in patients with acute ischemic stroke (AIS) caused by arterial occlusion in the anterior circulation (1–5). For patients with acute ischemia due to basilar artery occlusion, the rates of death or dependency are 76–78% (6), even when these patients are treated with intravenous or intra-arterial thrombolysis agents. Different case scenarios have shown that stent-retriever thrombectomy is a safe and feasible treatment technique in patients with acute basilar artery occlusion (BAO) (7–10). However, for acute intracranial atherosclerosis-related occlusion (ICASO), which is more common in Asia (11–14), salvage therapies (15), such as balloon angioplasty and stent angioplasty, are necessary to increase the rate of successful and complete reperfusion. The associated risks, such as perforator occlusion (16), endothelial damage (17, 18), microvascular dysfunction (19), and reocclusion of recanalized vessels (20–22), may cause intracranial hemorrhage or even worse outcomes.

Many studies have investigated methods to prevent the negative events caused by antithrombotic EVT, either in the anterior or the posterior circulation. A *post hoc* analysis from the Multicenter Randomized Clinical Trial of Endovascular Treatment for Acute Ischemic Stroke from the Netherlands reported that patients on a previous oral antiplatelet drug regimen were twice as likely to experience a favorable functional outcome (23). Injection of low-dose tirofiban combined with EVT can enhance recanalization rates (24–26). However, there have been no studies confirming which method is safer and/or more effective. The aim of the present study, therefore, was to compare outcomes in patients who underwent EVT plus tirofiban (EVT + tirofiban) therapy vs. those who underwent EVT in conjunction with oral antiplatelet drug therapy.

## Methods

### Study Design

**Figure 1:**
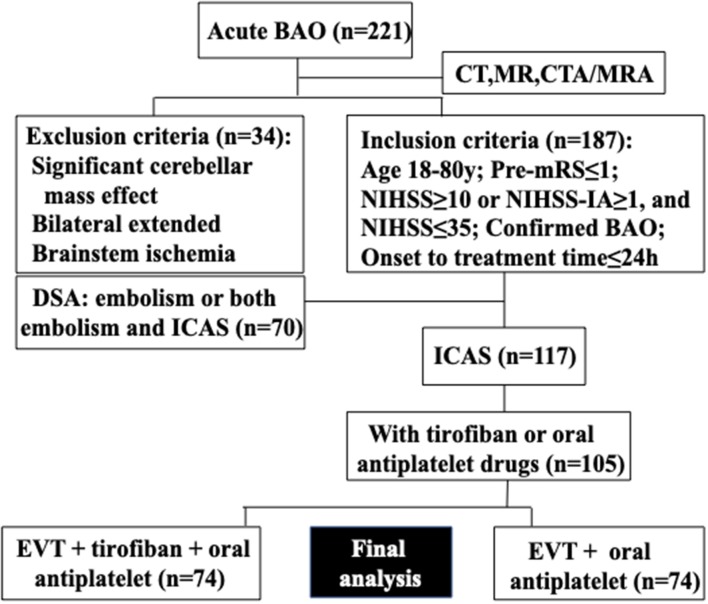
Flowchart of the patient selection.

Consecutive patients presenting with ICAS-related acute BAO who underwent EVT + tirofiban + oral antiplatelet or EVT + oral antiplatelet drug therapy at Beijing Tiantan Hospital (Beijing, China) between January 2012 and July 2018 were included in this retrospective study. Intravenous thrombolysis with tissue plasminogen activator was allowed prior to EVT, consistent with current guidelines (27). The details of patient selection and grouping are presented in Figure 1 according to previous studies (28–30). Tirofiban was considered for application in the following situations/parameters: (1) rescue treatment with emergency stenting for residual artery stenosis or failed thrombectomy; (2) rescue treatment with emergency balloon angioplasty for residual artery stenosis or failed thrombectomy; (3) local new thrombosis or vascular dissection of the responsible vessel; and (4) severe atherosclerosis lesions with a high possibility of reocclusion after being recanalized. Informed consent was obtained from all participants or their relatives, and the protocol was approved by the Institutional Review Board of Beijing Tiantan Hospital.

Plain computed tomography (CT) of the brain was performed first to exclude intracranial bleeding. Magnetic resonance imaging (MRI) was used to exclude large brainstem infarctions. CT angiography (CTA) or magnetic resonance angiography (MRA) was used to confirm the presence of acute BAO. The clinical and radiological data of these patients were retrospectively collected and analyzed and included age; sex; stroke risk factors, including hypertension, diabetes mellitus, hypercholesterolemia, and current smoking status; initial stroke severity, as reflected by the National Institutes of Health Stroke Scale (NIHSS) score; initial imaging modality; previous intravenous thrombolysis; type of EVT device(s); onset to puncture time; procedure duration; onset to recanalization time; and collateral status.

### EVT

The decision to perform EVT was made by a stroke neurologist and a neuroradiologist based on the CT, CTA, or MRA results of the patient. All EVT procedures were performed by a neurointerventionalist with sufficient experience in neurovascular intervention in mechanical thrombectomy for AIS (>50 cases). Cerebral angiography and EVT were performed under general anesthesia or conscious sedation after evaluation by a dedicated anesthesiology team. A stent retriever or aspiration was used first, with a possible switch to another strategy in the case of recanalization failure (mTICI grade = 0–2a) with the first approach. Underlying ICAS was determined when significant stenosis was seen on the initial diagnostic angiography or on follow-up angiography after a mechanical thrombectomy procedure (31). Causes of acute BAO were classified based on our previous research (32). ICAS was defined as significant fixed focal stenosis that could be resolved by means of angioplasty or stent insertion at the site of occlusion. Significant stenosis was defined as (1) fixed stenosis ≥70% or (2) fixed stenosis ≥50% in addition to either angiographical evidence of impaired perfusion or evidence of reocclusion after sufficient treatment with a stent retriever. The cause of acute BAO was classified as embolism based on the following: (1) after clot retrieval, there was no focal stenosis; (2) an embolus was removed with a stent retriever; or (3) MRA or CTA performed within 1 week after the procedure showed lack of stenosis in the responsible artery. In cases of recanalization failure (mTICI grade 0–2a after mechanical thrombectomy within 10 min), intracranial angioplasty or stenting was performed. All patients immediately received a loading dose of 300 mg of aspirin and 300 mg of clopidogrel orally. Patients with daily aspirin (100 mg) or/and clopidogrel (75 mg) medication before the intervention did not receive a loading dose. Alternatively, tirofiban therapy (Grand Pharmaceutical Co Ltd, China) was applied. Standard procedure was to administer 5 mg of tirofiban diluted in 100 ml of normal saline and infused with a 6- to 8-ml (0.3–0.4 mg) loading dose via a catheter at a rate of 1 ml/min. Intravenous tirofiban was continued at a rate of 0.15 μg/kg/min for the next 24 h. Intravenous tirofiban was continued at a rate of 0.15 μg/kg/min for the next 24 h. If there were no hemorrhagic complications seen on the head CT scan, aspirin and clopidogrel (one tablet each) were used with overlap for 4–6 h during the operation. If CT performed 24 h after the operation revealed no dynamic bleeding or hemorrhagic transformation, dual antiplatelet therapy was maintained for at least 3 months after the procedure, followed by lifelong aspirin or clopidogrel monotherapy thereafter.

### Image Interpretation

Diffusion-weighted images were assessed using the pons-midbrain index (using the posterior communicating artery) and the American Society of Interventional and Therapeutic Neuroradiology/Society of Interventional Radiology (ASITN/SIR) (29, 33) grading system. All images were analyzed retrospectively by two neurologists blinded to the patient information and study protocol. If no consensus was reached, a third neurointerventionalist with 20 years of experience made the final decision.

### Outcome Measures

For primary effectiveness, outcomes were assessed according to the BASICS definition (34). Functional independence and a favorable outcome were defined as mRS ≤ 2 and mRS ≤ 3, respectively. Other effectiveness outcome measures included the rates of successful recanalization [modified treatment for cerebral infarction (mTICI) = 2b−3] and complete recanalization (mTICI = 3) (35).

The safety outcome measures included 90 day mortality and rates of intracerebral hemorrhage and symptomatic intracerebral hemorrhage (SICH) within 24 h according to the criteria described in the European Cooperative Acute Stroke Study III (ECASS III) (36).

### Statistical Analysis

Study data were collected on standard forms, evaluated for completeness, and double keyed into an EpiData statistics data document. Statistical analysis was performed using R (http://www.R-project.org, The R Foundation for Statistical Computing, Vienna, Austria) and EmpowerStats (http://www.empowerstats.com, X&Y Solutions, Inc., Boston, MA, USA). Continuous variables are expressed as medians and interquartile ranges (IQRs) and as absolute numbers and percentages, while categorical variables are expressed as the means and standard deviations (SDs). Shapiro–Wilk test, histogram, and QQ chart were used to confirm normal distribution of data. The chi-squared test was used to compare categorical variables, *t*-tests were used to compare continuous variables, and the Mann–Whitney *U* test was used to compare scores. Logistic regression was performed to estimate odds ratios (ORs) for 90 day mortality and primary effectiveness outcomes (mRS ≤ 2 and ≤ 3). Variables including the NIHSS score, sex, standard preoperative intravascular tissue plasminogen activator, general anesthesia, stent thrombectomy, and intraoperative heparinization were entered into a logistic regression model. All tests were two-tailed, and statistical significance was determined at a *P* level of 0.05.

## Results

Of 105 patients, 71 [mean (± SD) age, 60.0 ± 8 years; 86.49% men] underwent EVT + tirofiban + oral antiplatelet drug therapy, whereas 34 (mean age, 60.0 ± 10 years; 83.87% men) underwent EVT + oral antiplatelet drug therapy.

A comparison of the baseline data and treatment procedures between the two groups is shown in Table 1. The NIHSS score was higher in patients who underwent EVT + tirofiban + oral antiplatelet drug therapy (25 [IQR 13–35] vs. 12 [IQR 10–23]; *p* = 0.005) than in those who underwent EVT + oral antiplatelet drug therapy (Table 1). More patients in the EVT + tirofiban + oral antiplatelet drug therapy group required stent thrombectomy (77.03% vs. 45.16%; *p* = 0.001) than patients in the EVT + oral antiplatelet drug therapy group, and fewer patients required intraoperative heparinization (40.54% vs. 70.97%; *p* = 0.004), which may have been related to differences in the severity of neurological deficits at the time of admission between the two groups.

**Table 1:** Comparison of baseline data and treatment procedures between patients who underwent ET +tirofiban + oral antiplatelet and those who underwent ET + oral antiplatelet drug therapy.

**Baseline characteristics, treatment procedures, and outcomes**	**Treatment**		***P*-value**
	**ET + Tirofiban+ oral antiplatelet** **(*n* = 74)**	**ET + oral antiplatelet drug (*n* =31)**	
Age, mean (SD)	60 (8)	60 (10)	0.944
Male sex (%)	64 (86.49%)	26 (83.87%)	0.764
NIHSS, median, points (IQR)	25 (13–35)	12 (10–23)	0.005
Door to needle, median, min (IQR)	420 (300–540)	450 (307–705)	0.165
Operative time, median, min (IQR)	90 (60–120)	60 (60–120)	0.224
Medical history			
Previous stroke	18 (24.32%)	7 (22.58%)	>0.999
Hypertension	54 (72.97%)	25 (80.65%)	0.446
Diabetes mellitus	19 (25.68%)	10 (32.26%)	0.485
Hyperlipidemia	19 (25.68%)	8 (25.81%)	>0.999
Previous smoking	30 (40.54%)	11 (35.48%)	0.628
Coronary heart disease	7 (9.46%)	2 (6.45%)	>0.999
Affected position			0.749
Proximal BA (including intracranial VA)	50 (67.57%)	23 (74.19%)	
Middle BA	23 (31.08%)	8 (25.81%)	
Distal BA	1 (1.35%)	0 (0.00%)	
Tandem occlusions	0 (0.00%)	1 (3.23%)	0.295
ASITN/SIR collateral score			0.262
Score of 0 or 1	28 (37.84%)	16 (51.61%)	
Score of 2	39 (52.70%)	11 (35.48%)	
Score of 3 or 4	7 (9.46%)	4 (12.90%)	
Standard IV rt-PA preoperative	18 (24.32%)	2 (6.45%)	0.054
General anesthesia	64 (86.49%)	22 (70.97%)	0.093
Stent thrombectomy	57 (77.03%)	14 (45.16%)	0.001
Intra-arterial thrombolysis	15 (20.27%)	10 (32.26%)	0.214
Intraoperative Heparinization	30 (40.54%)	22 (70.97%)	0.004
Intracranial angioplasty	62 (83.58%)	21 (67.74%)	0.125
Balloon angioplasty alone	15 (20.27%)	7 (22.58%)	
Stent implantation	47 (63.51%)	14 (45.16%)	

*Values are numbers with percentages in parentheses, unless indicated otherwise. IQR, interquartile range; NIHSS, National Institutes of Health Stroke Scale; SD, standard deviation; ASITN/SIR, American Society of Interventional and Therapeutic Neuroradiology/Society of Interventional Radiology*.

A comparison of higher recanalization rates, 90 day mortality, SICH, and functional outcomes between the two groups is summarized in Table 2. In both groups, there was a higher rate of successful recanalization (mTICI 2b-3) among patients who underwent EVT + tirofiban + oral antiplatelet drug therapy (93.24% vs. 77.42%; *p* = 0.038). After comparing the two groups, the functional independence outcome rate (mRS 0 to ≤ 2) at 90 days (32.43% vs. 51.61%, *p* = 0.065), 7 day intracerebral hemorrhage (16.22% vs. 6.45%, *p* = 0.223), 90 day mortality (16.22% vs. 9.68%, *p* = 0.544), 7 day SICH (4.05% vs. 0.00%, *p* = 0.553), and 30 day responsible artery reocclusion (16.22% vs. 12.90%, *p* = 0.773) was not significantly different. The favorable outcome rate (mRS 0 to ≤ 3) in the EVT + tirofiban + oral antiplatelet drug therapy group was higher than that in the EVT + oral antiplatelet drug therapy group, owing to the differences in severe neurological deficits at admission.

**Table 2:** Comparison of higher recanalization rates, mortality at 90 days, symptomatic intracerebral hemorrhage (SICH), and function outcomes between EVT + tirofiban + oral antiplatelet and EVT + oral antiplatelet drug therapy.

**Outcome measures**	**Treatment**		***P* value**
	**ET + tirofiban+ oral antiplatelet** **(*n* = 74)**	**ET + oral antiplatelet drug (*n* =31)**	
**Angiographic outcome (mTICI)**			
2b-3	69 (93.24%)	24 (77.42%)	0.038
Good Outcome (mRS 0– ≤ 2)	24 (32.43%)	16 (51.61%)	0.065
mRS 0– ≤ 3	35 (47.30%)	22 (70.97%)	0.026
90 days mortality	12 (16.22%)	3 (9.68%)	0.544
7 days intracerebral hemorrhage	12 (16.22%)	2 (6.45%)	0.223
7 days SICH	3 (4.05%)	0 (0.00%)	0.553
30 days responsible artery reocclusion	12 (16.22%)	4 (12.90%)	0.773

Values are numbers with percentages in parentheses, unless indicated otherwise.

*mRS, modified Rankin Scale; mTICI, modified Thrombolysis in Cerebral Infarction; SICH, symptomatic intracerebral hemorrhage, tPA, tissue plasminogen activator*.

Adjusted logistic regression analysis revealed that treatment with EVT + tirofiban + oral antiplatelet drug therapy was associated with an increased rate of successful recanalization (OR 0.18 [95% confidence interval (CI) 1.24–24.39]; *p* = 0.025), whereas no differences were found in SICH (OR 0.00 [95% CI 0.00–Inf]; *p* = 0.998), 90 day mortality (OR 1.19 [95% CI 0.17–4.05]; *p* = 0.826), or functional independence outcomes (mRS 0 to ≤ 2) (OR 1.43 [95% CI 0.23–2.17]; *p* = 0.538) between the groups (Table 3).

**Table 3:** Univariate and multivariate logistic regression analysis.

**Outcome measures**	**Univariate Logistic** **Regression Analysis**	**Multivariate Logistic** **Regression Analysis**
**Angiographic outcome (mTICI 2b-3)**		
ET + tirofiban + oral antiplatelet	1.0	1.0
ET + oral antiplatelet	0.25 (0.07, 0.86) 0.028	0.18 (0.04, 0.81) 0.025
**Good Outcome (mRS 0–≤** **2)**		
ET + tirofiban + oral antiplatelet	1.0	1.0
ET + oral antiplatelet	2.22 (0.94, 5.23) 0.068	1.43 (0.46, 4.42) 0.538
**mRS 0–≤** **3**		
ET + tirofiban + oral antiplatelet	1.0	1.0
ET + oral antiplatelet	2.72 (1.11, 6.70) 0.029	1.45 (0.48, 4.35) 0.509
**90 days mortality**		
ET + tirofiban + oral antiplatelet	1.0	1.0
ET + oral antiplatelet	0.55 (0.14, 2.12) 0.388	1.19 (0.25, 5.77) 0.826
**7 days intracerebral hemorrhage**		
ET + tirofiban + oral antiplatelet	1.0	1.0
ET + oral antiplatelet	0.36 (0.07, 1.70) 0.195	0.59 (0.11, 3.31) 0.553
**7 days SICH**		
ET + tirofiban + oral antiplatelet	1.0	1.0
ET + oral antiplatelet	0.00 (0.00, Inf) 0.996	0.00 (0.00, Inf) 0.998
**30 days responsible artery reocclusion**		
ET + tirofiban + oral antiplatelet	1.0	1.0
ET + oral antiplatelet	0.77 (0.23, 2.59) 0.667	0.56 (0.14, 2.24) 0.409

Data: OR (95% CI) P-value.

*mRS, modified Rankin Scale; mTICI, modified Thrombolysis in Cerebral Infarction; SICH, symptomatic intracerebral hemorrhage; tPA, tissue plasminogen activator*.

## Discussion

ICAS occlusion is more common in the posterior than in the anterior circulation, especially among the Asian population (22, 37). Therefore, rescue therapies such as emergency angioplasty or stenting are often required when treating these patients with EVT (15, 21), thereby inducing the removal of *in situ* thrombi (17), instability in the ICAS endothelium (38), and the “snowplow” effect (21). These steps result in perforator occlusion, microvascular dysfunction, and reocclusion of recanalized vessels. Many treatment strategies, including EVT + tirofiban + oral antiplatelet drug therapy (23) and EVT + oral antiplatelet drug therapy (24, 25), have been performed on patients with anterior and posterior circulation infarction in previous studies to prevent these drawbacks.

Due to the disparate characteristics of patients with posterior circulation cerebral infarction, intravascular tirofiban is more convenient than oral antiplatelet drug therapy. First, most patients with posterior circulation infarction exhibit consciousness disorders and difficulty swallowing. Oral antiplatelet drugs need to be administered through a gastric tube, the insertion of which is time-consuming. Second, it is important to differentiate an ICAS occlusion from an intracranial embolism before operating on patients without a clear history of intracranial artery stenosis. For these patients, intravascular tirofiban has the advantage of a rapid onset. Therefore, the present study compared the safety and outcomes in patients who underwent EVT+ tirofiban drug therapy vs. those who underwent EVT + oral antiplatelet drug therapy.

Our study found that acute intracranial atherosclerosis-related vertebrobasilar artery occlusion treated with EVT + tirofiban + oral antiplatelet drug therapy was associated with an increased rate of successful recanalization (OR 0.18 [95% (CI) 1.24–24.39]; *p* = 0.025), whereas no differences were found in SICH (OR 0.00 [95% CI 0.00–Inf]; *p* = 0.998) or 90 day mortality (OR 1.19 [95% CI 0.17–4.05]; *p* = 0.826). Functional independence outcomes at 90 days (mRS 0 to ≤ 2) were not significantly different (OR 1.43 [95% CI 0.23–2.17]; *p* = 0.538), which may be attributed to the severity of the neurological deficits between the groups at the time of admission and the small sample size. Since the investigation was an observational one based on a single-center prospective registry study, there are several limitations. First, the administration route, dose, and duration of tirofiban used in this study were pragmatic. Furthermore, inherent bias because of the retrospective and monocentric study design was inevitable. Compared to patients with EVT + oral antiplatelet drug therapy, those with EVT + tirofiban + oral antiplatelet drug therapy had a higher NIHSS score at baseline and a more frequent application of stent thrombectomy and intraoperative heparinization. However, after adjusting for potential confounders and conducting a multivariate analysis, we presume that this bias probably had little influence on our results. A multicenter study involving a larger sample size or a randomized controlled trial is needed to verify this result.

## Conclusions

In summary, for patients who have trouble swallowing and those without a clear history of intracranial artery stenosis, intravascular tirofiban treatment can be performed to save time. Our results demonstrated that EVT + tirofiban + oral antiplatelet drug therapy was not superior to EVT + oral antiplatelet drug therapy in terms of the functional independence outcomes at 90 days. Therefore, a more effective perioperative administration route, time, and dosage await further study.

## Data Availability Statement

The datasets generated for this study are available on request to the corresponding author.

## Ethics Statement

All procedures performed in studies involving human participants were in accordance with the ethical standards of Beijing Tiantan Hospital, Capital Medical University medical ethics committee and with the 1964 Helsinki declaration and its later amendments or comparable ethical standards.

## Author Contributions

ZM designed the research and wrote the manuscript. XS and HZ designed the research. XS, HZ, and ZM performed the research. XT, FG, and GM analyzed the data.

### Conflict of Interest

The authors declare that the research was conducted in the absence of any commercial or financial relationships that could be construed as a potential conflict of interest.

## Abbreviations

EVT: endovascular treatment
SICH: symptomatic intracranial hemorrhage
AIS: acute ischemic stroke
ICASO: intracranial atherosclerosis-related occlusion
tPA: tissue plasminogen activator
CT: computed tomography
CTA: computed tomography angiography
MRI: magnetic resonance imaging
MRA: magnetic resonance angiography
NIHSS: National Institutes of Health Stroke Scale
mTICI: modified Thrombolysis in Cerebral Infarction
mRS: modified Rankin Scale
ORs: odds ratios.
